# COVID‐19 pandemic and socio‐environmental inequality: A narrative review

**DOI:** 10.1002/hsr2.1372

**Published:** 2023-06-27

**Authors:** Laleh R. Kalankesh, Zahed Rezaei, Ali Mohammadpour, Mahmoud Taghavi

**Affiliations:** ^1^ Social Determinants of Health Research Center Gonabad University of Medical Sciences Gonabad Iran

**Keywords:** air pollution, COVID‐19, economics, education, inequality, safe water, socio‐environmental status, waste management

## Abstract

**Background and Aims:**

The COVID‐19 pandemic has provided preliminary evidence of the existence of health, social, and environmental inequalities. This inequality encompasses inadequate access to safe water, clean air, and wastewater management, as well as limited socioeconomic and educational opportunities. These issues have not received sufficient attention during the pandemic. The purpose of this narrative review is to provide a comprehensive summary and analysis of the existing literature on a specific topic, ultimately leading to a conclusion based on the evidence presented.

**Methods:**

The search methodology for this study involved conducting comprehensive searches of scientific databases, including PubMed, ScienceDirect, LILACS, and Google Scholar, from 2019 to 2023. The study focused on a specific theme and its relevant aspects related to global environmental health and society. Keywords such as COVID‐19, inequities, and environmental health were used for searching. Additionally, the Boolean operator “AND” was used to combine these descriptors.

**Results:**

Unequal exposure to air pollution has been reported in Africa, as well as in large parts of Asia and Latin America, according to the data that has been obtained. The pandemic has also resulted in a surge in healthcare waste generation, exacerbating the environmental impact of solid waste. Furthermore, there is evidence indicating significant disparities in the severe lack of access to sanitation services between developing nations and low‐income regions. The issues related to water availability, accessibility, and quality are subject to debate. It has been reported that SARS‐CoV‐2 is present not only in untreated/raw water, but also in water bodies that act as reservoirs. Moreover, insufficient education, poverty, and low household income have been identified as the most significant risk factors for COVID‐19 infection and mortality.

**Conclusion:**

It is evident that addressing socio‐environmental inequality and striving to narrow the gap by prioritizing vulnerable populations are imperative.

## 1 INTRODUCTION

The COVID‐19 pandemic has highlighted global disparities in social, environmental, and health factors. Although different aspects of health inequities have been highlighted during the pandemic, it has been strongly emphasized that environmental factors play a key role in the extent and prevalence of COVID‐19 infections. However, previous studies have identified a significant gap in this area of research.^1^ Furthermore, the occurrence of COVID‐19 infections can be influenced by various environmental and social factors, which can affect the level of exposure, mortality rates, and recovery rates. The undeniable impact of unequal environmental exposure, including air, water, and wastewater sources, on the severity of the COVID‐19 pandemic has been extensively documented. The static mortality rates observed across different regions of the world appear to be linked to differential levels of exposure to environmental pollutants. These pollutants are often associated with disparities in socioeconomic status (SES).^2^ The management of safe water and wastewater systems, as well as the availability of clean air, are crucial factors in preventing the spread of COVID‐19.

### 1.1 Basic discussion

The report by Acharya et al. indicates that the slow spread of COVID‐19 and insufficient hospital capacity have led to an increase in home care, resulting in a significant amount of waste contaminated with the virus. This has caused disruptions in the management of municipal solid waste.^3^ Furthermore, Chirani et al. have determined that the increased waste produced by infected individuals and hospitals can lead to a deterioration in water quality, which could potentially serve as a means of virus transmission.^4^ Additionally, social factors such as cultural context, family dynamics, financial constraints, lifestyle choices, and dietary habits also play a significant role. The increasing number of COVID‐19 cases is having a disproportionate impact on individuals living in communities with weakened immune systems, such as those in impoverished areas. The pandemic has raised concerns about the higher mortality and hospitalization rates experienced by communities that are crowded and impoverished. The containment of the virus in these communities presents significant challenges and may even be considered unfeasible.^5^ It is noteworthy that disparities are not solely encountered in obtaining essential amenities in nations with middle and low‐income levels, but also in confronting inherent inequalities such as living in extreme temperatures, abnormal weather patterns, and atmospheric conditions.^6^ In many regions of Asia and Africa, social inequality persists significantly. Additionally, a significant portion of the population lives in unstable housing and unsanitary environments, with limited and inconsistent access to water and sanitation facilities. Furthermore, the World Health Organization has documented the emergence of new strains of the COVID‐19 virus globally. The Beta variant (B.1.351) was first identified in South Africa, while the Gamma variant (P.1) was initially detected in travelers from Brazil to Japan. The Delta variant (B.1.617.2) was first identified in India. Furthermore, there has been a significant surge in COVID‐19 cases across Southeast Asian nations, with a total of 11,324,390 confirmed cases and 249,529 fatalities reported so far. As of January 2022, the Middle East and North Africa (MENA) region had recorded more than 30 million COVID‐19 cases and over 370,000 deaths.

### 1.2 Indication of purpose

Undoubtedly, there has been marked increase in mortality rates in low‐income nations that host a substantial number of refugees, such as Lebanon and Jordan. The accurate determination of mortality rates linked to the virus in the most conflict‐ridden countries in the region, particularly Syria, Libya, and Yemen, is unattainable. Undoubtedly, the issue of environmental degradation has become increasingly prominent, particularly in the context of the ongoing pandemic. A growing body of scholarly research has been dedicated to exploring the relationship between environmental factors and the COVID‐19 pandemic.^7^ A comprehensive analysis of the impact of environmental and social inequalities during a pandemic outbreak has not been conducted yet. It is crucial to identify and emphasize the various factors associated with social and environmental inequalities that exist in middle‐ and low‐income countries. This will simplify the design of development strategies and policies aimed at avoiding and resolving socio‐environmental inequalities, thereby reducing the impact of the pandemic on the most vulnerable populations. The current study provides a summary of the social and environmental inequalities that have arisen during the pandemic. These inequalities have the potential to influence the severity of both the prevalence and mortality of cases. The article delves into the adverse consequences of environmental inequities, such as insufficient water, air, and waste management. It also explores the influence of social inequities, such as education and economic disparities, on the spread and mortality rate of the pandemic.

## 2 METHOD

The present study has received ethical approval from the appropriate research committee (IR.GMU.REC.1401.124). The primary objective of this narrative review is comprehensively summarize the current literature on a specific subject matter, with the ultimate goal of drawing evidence‐based conclusions. The search methodology for this study involved comprehensive searches of scientific databases such as PubMed, ScienceDirect, LILACS, and Google Scholar from 2019 to 2023. The study focused on a specific theme and its relevant aspects related to global environmental health and society. Keywords such as COVID‐19, inequities, and environmental health were used for searching. Additionally, the Boolean operator “AND” was used to combine these descriptors. The study employed the nonsystematic literature review method guided by Pereira et al.^8^

## 3 ENVIRONMENTAL INEQUITIES ASPECT

**Figure 1:**
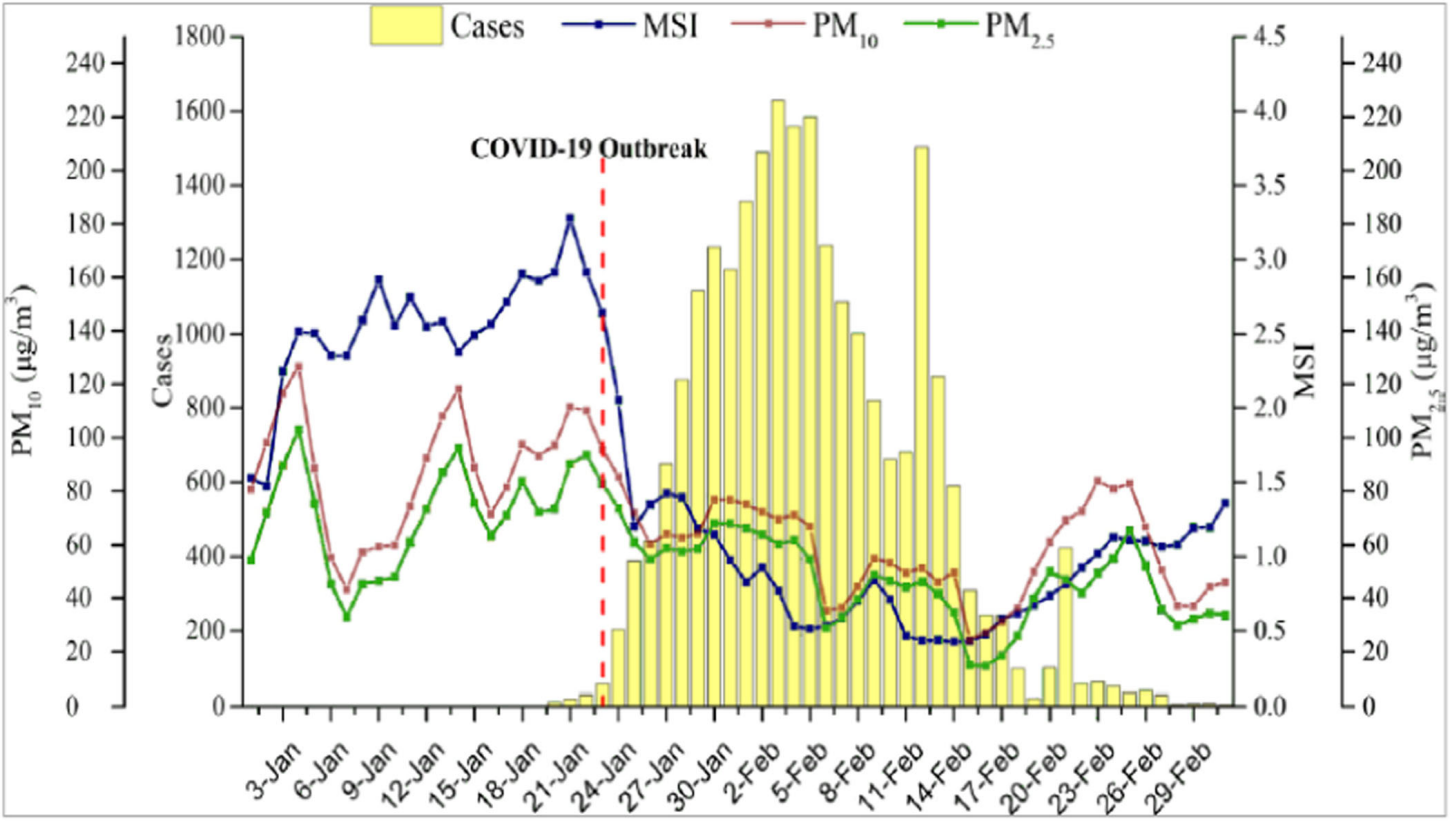
Trends of daily particle matter (PM) levels, mobility scale index (MSI), and confirmed COVID‐19 cases in 63 cities of China.^11^

The environmental situation varies across different regions, and the impacts of COVID‐19 are not uniform for all populations. Nonetheless, it is essential to prioritize environmental justice issues, such as ensuring access to clean air, safe water, and effective wastewater treatment systems, even during the pandemic (Figure 1). The question arises about whether individuals living in unhealthy environmental conditions are more vulnerable to infections compared to those residing in healthier environments. The aforementioned statement suggests that the prevalence of the disease is unevenly distributed among developing countries, particularly in Africa and certain regions of Asia where access to healthcare and basic necessities of life are not guaranteed. There is a clear correlation between certain environmental injustices, such as living in heavily polluted areas, inadequate housing, and overcrowding, and increased mortality rates due to COVID‐19. Furthermore, the ongoing global health crisis has emphasized the critical importance of ensuring widespread access to effective hand hygiene practices. Furthermore, research indicates significant disparities among vulnerable children and households that are disproportionately affected by the lack of access to clean water during the pandemic. Additionally, significant differences in the management of waste and water resources were observed among countries during the COVID‐19 pandemic.

### 3.1 Unhealthy air quality inequities

The World Health Organization (WHO) has reported that a significant proportion of the global population lives in areas that do not meet the organization's air pollution guidelines. Please provide a valid input. It is important to note that this not only pertains to air pollution, but also encompasses atypical weather conditions. Scholars have been investigating the potential links between air quality and the risks associated with COVID‐19 in light of the pandemic. However, there is a dearth of information in the literature regarding disparities in air quality. It has been suggested that higher rates of COVID‐19 deaths may be linked to unequal exposure to environmental hazards. Furthermore, the correlation between poor air quality and adverse effects of COVID‐19 has been notably significant during the outbreak.^9^ The unequal distribution of air conditioning resources has resulted in a disproportionate impact on the mortality rate of individuals residing in areas with poor air quality and limited financial means.^10^

### 3.2 Atmospheric pollution and COVID‐19 mortality

Moreover, the currently available COVID data indicates a correlation between prolonged exposure to air pollution and increased mortality rates during the pandemic. Furthermore, an increasing amount of evidence emphasizes a strong correlation between air quality and persistent hospitalization or mortality. The data presented in Figure 1 demonstrate that the onset of the COVID‐19 outbreak in 63 Chinese cities occurred on January 23, peaked on February 3, and subsequently declined, ultimately approaching zero by February 28. Notably, the daily average levels of PM10 and PM_2.5_ followed a similar pattern, both exhibiting a significant decrease after January 23 (Figure 1).

A recent investigation conducted in 140 urban centers throughout China has revealed that a significant proportion of confirmed cases of coronavirus, amounting to 70%, were linked to cities characterized by elevated levels of pollution.^12^ Similarly, a research investigation was conducted to examine the relationship between prolonged exposure to NO_2_ and the incidence of COVID‐19 cases in 66 administrative regions across Italy, Spain, France, and Germany. The findings reveal that out of the 4443 recorded deaths, 3487 (78%) were concentrated in the five regions with the highest levels of NO_2_, specifically in northern Italy and central Spain.

### 3.3 Disparity clean air availability

**Figure 2:**
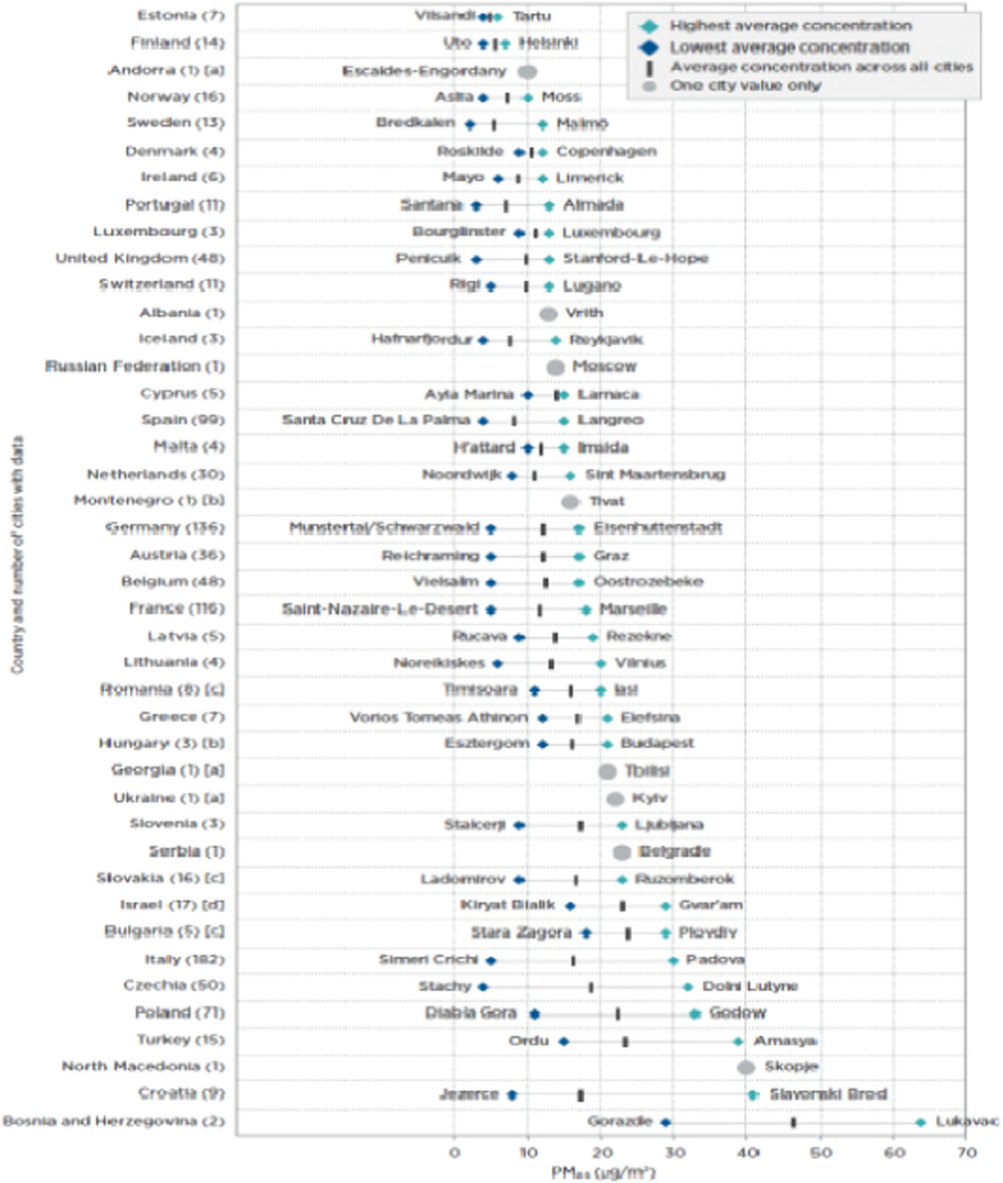
Average particle matter (PM_2.5_) concentration in various cities globally.^14^

An analysis of the global situation indicates that there is a disparity in the availability of clean air, which has been shown to be beneficial in the context of the COVID‐19 pandemic. On the other hand, the onset of the industrial era has resulted in significant discharge of pollutants caused by human activities. This has had a widespread impact on a global level, with the most vulnerable communities and nations being disproportionately affected, especially those with limited economic resources. Figure 2 illustrates the disparities in particle matter emissions across different nations, with certain countries exhibiting lower averages while others display elevated levels (Figure 2).^13^ The data presented in the figure indicate that particle matter emissions are not uniform across countries. Notably, the city of Lithonia, Georgia in the United States has the highest level of emissions recorded. All countries are impacted by these emissions, although developing nations may be more vulnerable. It is noteworthy that Jezerce, compared to other Albanian cities, has the lowest level of emissions.

Moreover, it is frequently observed that the most economically disadvantaged communities do not benefit from global industrialization, while the harmful consequences of pollution significantly affect their health and well‐being, especially in the context of the current pandemic.^15^ The COVID‐19 pandemic has exacerbated pre‐existing disparities, particularly in the Mediterranean regions. The intersection of varying degrees of exposure to air pollution, environmental injustices, and the ongoing pandemic poses a triple threat to underprivileged communities. This threefold danger requires significant examination. Based on statistical data gathered from Africa, Asia, and Latin America, it has been established that these regions continue to experience high levels of environmental air pollution compared to other populations. The unequal distribution of sources and mechanisms for ambient air pollutants has been a subject of debate. Certain areas within the region are more vulnerable to environmental shocks and lack community resources that could help alleviate the effects of climate change. The lack of vegetation that aids in air purification and cooling during heat waves exacerbates the problem. It is imperative to acknowledge the undeniable influence of historical inequity on the significant incidence of COVID‐19.

## 4 WAST MANAGEMENT AND RECYCLING INEQUITIES

The provision of sanitation is considered a fundamental human entitlement. In nations with limited financial resources and inadequate sanitation infrastructure, the most vulnerable segments of society disproportionately shoulder the burden of socioeconomic inequalities and insufficient sanitation facilities during pandemics and disease outbreaks. Insufficient sanitation pertains not only to substandard household sanitation facilities but also to inadequate waste management.^16^ Managing waste in the least developed countries presents a significant challenge during the global pandemic, especially in terms of ensuring safety. Notably, the latest data shows that significant proportion of healthcare services have inadequate management of healthcare waste, particularly in terms of safety.

### 4.1 Hospital waste management and COVID‐19 viruses

Additionally, the increase in healthcare waste during the pandemic worsens the immediate environmental impact. The proper disposal of significant amounts of infectious healthcare waste has emerged as a significant challenge. Inadequate waste management, particularly the handling of biomedical waste (BMW), has the potential to facilitate the transmission of viruses among the general population. The increase in the production of waste at a worldwide level has led to significant changes in the processes related to the generation and control of waste, including both medical and nonmedical waste. Given the current pandemic situation, it is crucial to implement effective waste management strategies to mitigate the negative socioeconomic and environmental impacts of the pandemic.^17^

### 4.2 Waste management discrepancy in low‐ and high‐income nations

**Figure 3:**
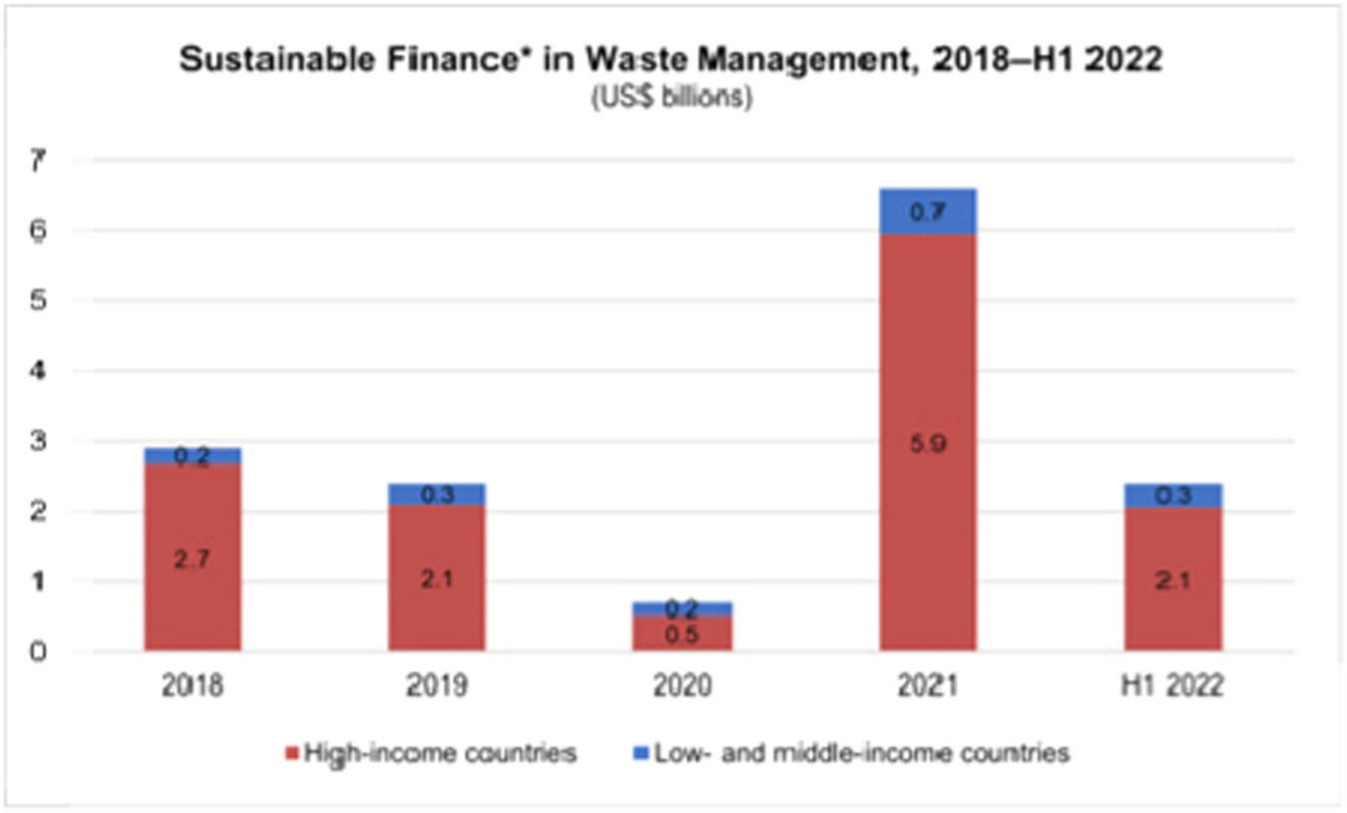
Trend of sustainable finance in high‐ and low‐income countries from 2018 to 2022.^20^

Developed nations have implemented extensive guidelines and protocols for the effective management of BMW. Conversely, it has been observed that developing and low‐income countries often lack sufficient waste management procedures, as illustrated in Figure 3. The data depicted in Figure 3 highlights the fact that economically viable waste management strategies have been increasingly adopted in affluent regions from 2018 to 2021. Furthermore, the data indicates that waste management achieved its highest level of financial sustainability in 2021 (5.6), while the lowest was recorded in 2020 (0.5). Despite the fact that the poverty rate in underprivileged nations has remained relatively low, at below 0.3, it reached its peak in 2021 (5.6). An efficient waste management infrastructure, which includes appropriate disposal methods for BMW, is imperative in mitigating environmental pollution and safeguarding groundwater and water resources. The World Health Organization (WHO) emphasizes the importance of implementing effective sanitation measures, adhering to proper hygiene practices, managing waste appropriately, and ensuring adequately equipped healthcare facilities to effectively curb the spread of viruses during the COVID‐19 pandemic.^18^ According to recent findings, there is a noticeable disparity in the availability of adequate sanitation facilities between individuals living in developing nations and those in low‐income regions.^19^

In several Latin American nations, such as Brazil, Cuba, and Colombia, a significant portion of rural communities lack adequate waste disposal infrastructure. Amidst the ongoing pandemic, Georgia, Moldova, and Ukraine have formed partnerships with the European Union to tackle waste management issues. Notably, certain countries, such as Armenia, Azerbaijan, and Belarus, have made noteworthy strides in this domain.^21^

## 5 ACCESS TO SAFE WATER

The issue of water security is currently a topic of increasing concern and complexity. The provision of safe drinking water and sanitation is closely associated with the well‐being of society. However, the accessibility of these crucial resources is influenced by disparities related to factors such as geography, economics, and social conditions.^22^ In line with the sustainable development goal (SDG) agenda for 2030, it is crucial to ensure that every individual has reasonable access to affordable and safe drinking water, as well as adequate and equitable sanitation facilities.^23^ Similarly, the WHO emphasizes the importance of providing justifiable and sustainable access to safe drinking water. Inadequate access to water may lead to adverse health effects, particularly for those who experience disadvantage or social exclusion. According to estimates by the WHO, almost 18% of disease outbreaks are associated with exposure to water pathways. The simultaneous presence of a high prevalence of infectious diseases and limited access to safe water for extended periods can lead to negative health consequences. The effective control of the virus requires individual isolation, regular hand washing, and efficient healthcare facilities.^24^ Access to safe water plays a crucial role in controlling the transmission of the coronavirus and promoting community health during a pandemic.

### 5.1 Disparities in access safe water in pandemic time

**Figure 4:**
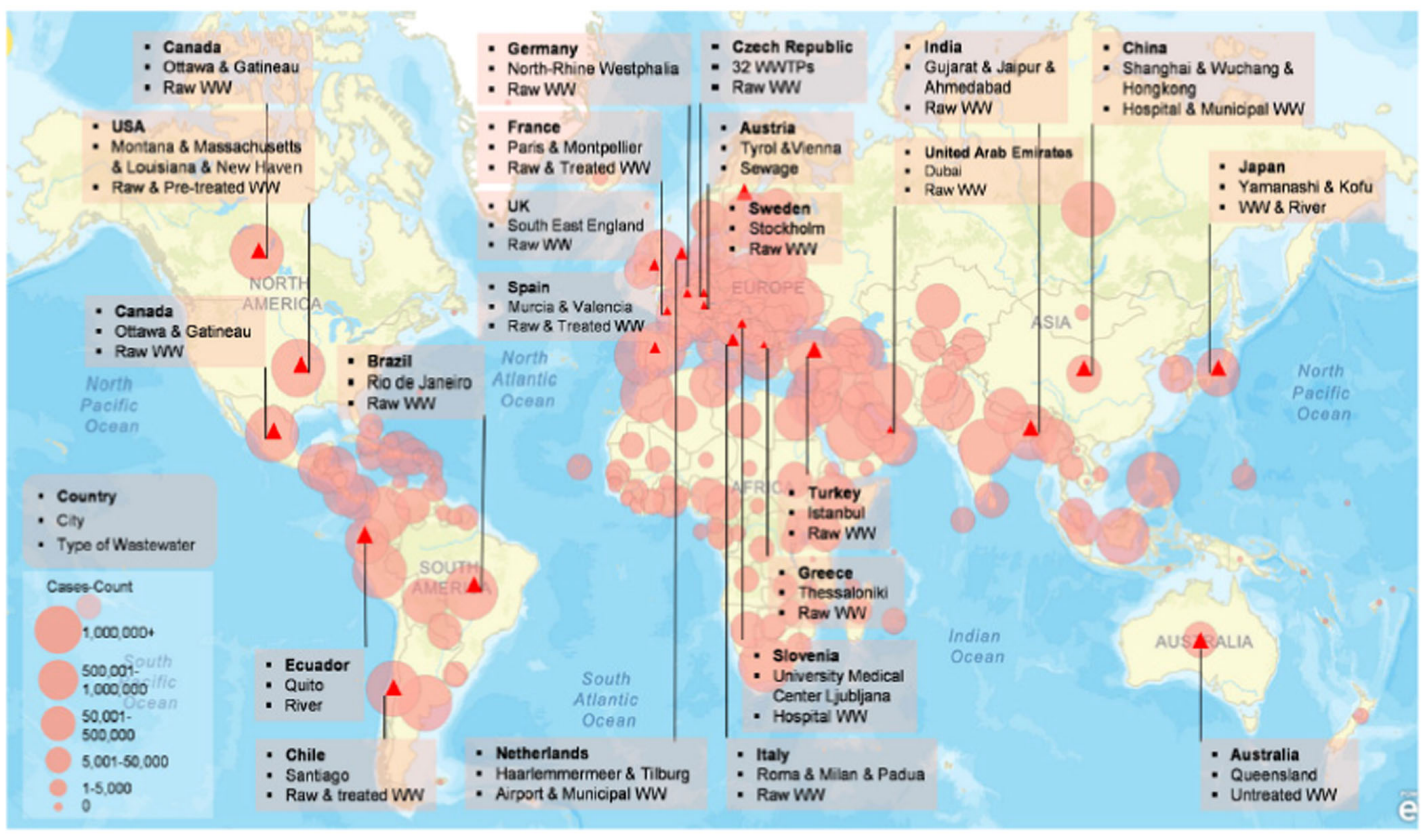
A review of confirmed cases SARS‐CoV‐2 in global water systems.^29^

Disparities in sanitation facilities, especially in terms of access to safe water, are influenced by geographical, economic, and social factors.^25^ The final report by the WHO indicated that an individual's living conditions could determine their risk of poor health and premature mortality.^26^ In the context of the pandemic, differences in mortality rates are observed between low‐income and high‐income societies. A common perspective suggests that handwashing is crucial for maintaining hygiene, but it can be a challenging task in areas where water is scarce. It has been established that the spread of the virus has exacerbated water scarcity in various regions across the globe, including Africa and Latin America, among others. The consequences of high population density in urban areas cannot be disregarded. These areas are often characterized by cramped living conditions, insufficient sanitation, and restricted access to water supply. wastewater‐based epidemiology (WBE) has been extensively implemented in various countries around the world, including Spain, Italy, the United Kingdom, the Netherlands, the United States, India, Japan, Germany, Australia, and the United Arab Emirates. However, there is a lack of published data on the use of WBE in developing countries.^27^ In contrast, developing nations may lack sufficient water and wastewater treatment infrastructure, as well as monitoring services for drinking water, which could potentially serve as a vulnerable surveillance system and early warning mechanism during a pandemic.^28^ However, the availability, accessibility, and quality of water present significant challenges for both developed and developing countries during a pandemic. A review of data from more than 20 countries has revealed the presence of SARS‐CoV‐2 in raw and untreated wastewater, as well as in secondary treatment and sewage sludge runoff. The virus has even been detected in rivers (Figure 4).^29^ Bhowmick and colleagues have documented the virus's ability to infiltrate through various pathways. The urban water cycle is particularly vulnerable to the impact of the water environment, as the SARS‐CoV‐2 virus can be detected in the fecal matter, urine, or vomits of an infected individual, and subsequently enter the sewer system. However, there are currently limited studies available on the persistence of SARS‐CoV‐2 in water and wastewater.^30^ Although the detection of coronavirus in water bodies is rare, it has been reported in the aquatic environments of rivers in Italy and Ecuador. However, the level of virus infectivity in these environments is still a subject for further discussion.

## 6 SOCIAL INEQUALITIES

The current complex social crisis poses a significant challenge to humanity. The pandemic has led to a global increase in social inequalities across various domains. Amidst the pandemic, people living in geographically disadvantaged areas are facing significant social inequalities on a global scale. In a pandemic scenario, the issues of escalating poverty, inequitable access to education, and healthcare pose a grave threat to a substantial proportion of the global population, particularly in low‐income countries such as those in sub‐Saharan Africa and Southern Asia.^31^ The overwhelming evidence suggests that a substantial portion of the world's population, around 4 billion people, live below a certain monetary threshold per day. This leads to a higher incidence of relative poverty and corresponding patterns of social exclusion. It is noteworthy that the COVID‐19 pandemic has been associated with social disparities in various areas, such as incidence, testing, severity, differential exposure to the virus, increased susceptibility to infection, and a higher prevalence of comorbidities.^32^ Furthermore, the COVID‐19 pandemic's varying effects on different social groups, such as unequal access to healthcare and education, economic disparities, social class, and race/ethnicity, are frequently disregarded, suppressed, or made invisible.^33^ The pandemic has shed light on social inequalities and underscored the short‐term and long‐term effects of the pandemic on these inequalities. While government response programs may alleviate some of the short‐term disparities associated with the pandemic, such as setbacks in education and human development or prolonged unemployment, they may not fully address the underlying issues.^34^ The impact of COVID‐19 on intercountry and intra‐country inequality will also be analyzed. Moreover, the lockdown has led to the emergence of novel forms of inequality. These include discrepancies in health safety measures between individuals who can work from home and those who are required to be physically present at their workplace. Additionally, families living in cramped apartments that are not conducive to outdoor activities or exercise are facing substandard living conditions. Moreover, addressing digital insecurity, such as limited network coverage, inadequate learning opportunities, and unregulated pricing, is crucial to ensure genuine digital equality.^35^

## 7 ECONOMIC INEQUALITY

The probability of contracting and succumbing to the virus can be significantly influenced by economic inequality. The impact of the economic factor on the outcomes is not uniform. The virus's disproportionate impact is worsened by inadequate healthcare infrastructure in developing communities and noncompliance with physical distancing measures. Moreover, societies with low SES are at a higher risk of infection and mortality. These societies are often characterized by substandard housing conditions, poverty, and overcrowded households.^36^ The availability of the COVID‐19 vaccine is affected by socioeconomic disparities. Recent studies have shown that socioeconomic inequalities have a negative impact on COVID‐19 testing and incidence rates. In regions heavily affected by the virus, testing rates have decreased in the most economically disadvantaged areas. Additionally, research indicates that individuals in lower socioeconomic groups have limited access to protective measures.^37^ Unexpected findings have emerged, revealing that individuals with higher median incomes have a higher incidence of infection, mortality, or confirmed diagnosis. Some evidence has produced contradictory results, suggesting that regions with higher income^38^ or lower poverty rates^38^ may have a higher frequency of infection. Furthermore, research has shown a significant correlation between poverty and enrollment in Medicaid, which has been linked to higher mortality rates in different parts of the United States. Furthermore, a study has shown that individuals in the working age group who earn the lowest income face a 5.4 times greater risk of mortality from coronavirus compared to those with the highest income.^39^ Moreover, deprivation has been identified as a strong predictor of COVID‐19 mortality. Research has shown that a one percentage point increase in deprivation is associated with a 5% increase in mortality.^40^ Similarly, research has shown that individuals from low‐income populations are 3.6 times more likely to be admitted to the ICU.^41^ However, there is a concerning trend of increasing COVID‐19 mortality rates among African Americans/Blacks and Hispanics, as well as among unemployed individuals. The mortality rates for these groups are 5.08%, 4.5%, and 4%, respectively.^42^ Nine studies have analyzed 12 correlations between individual SES and inequality, with results indicating that low SES groups are disproportionately affected (Table 1).

**Table 1:** Association between socioeconomic inequalities and COVID‐19.

Indicator	Study	Country	Result
(A) Regional socioeconomic indicators			
Regional income	Price‐Haywood et al.^43^	USA	Higher hospitalization risk correlates with lower income
	Azar et al.^44^		
	Mollalo et al.^45^	USA	
	Chow et al.^46^	Germany	Higher incidence correlates with lower income
	Pluemper and Neumayer^47^		
	Abedi et al.^48^		
	Li et al.^49^	USA	Higher incidence correlates with higher income
	Mukherji et al.^50^	Germany	
	Pluemper and Neumayer^47^		
	Vahidy et al.^51^	USA	Lower incidence correlates with higher income
	Sy et al.^52^		
	Takagi et al.^53^	USA	No correlation between prevalence and income
Regional income inequality	Mollalo et al.^45^		Higher incidence correlates with greater inequality
	Mukherji^50^	USA	Higher incidence correlates with greater inequality
	Mukherji^50^		Higher mortality correlates with greater inequality
Regional poverty rate	Ramirez and Lee^54^		
	Wadhera et al.^57^	USA	Higher mortality correlates with greater poverty
	Abedi et al.^48^	UK	
	Cyrus et al.		
	Chen and Krieger^55^		
	Rose et al.^56^		
	Wadhera et al.^57^	USA	Higher hospitalization risk correlates with greater poverty
	Takagi et al.^53^	USA	No correlation between mortality and poverty
	Guha et al.^58^		No higher mortality with greater poverty
	Li et al.^11^		
	Chen and Krieger^55^	USA	Higher incidence correlates with higher poverty
	Takagi et al.^53^	USA	Higher prevalence correlates with greater poverty
	Takagi et al.^53^		
(B) Individual socioeconomic indicators			
Income	Okoh et al.^59^	USA	
	Okoh et al.^59^	USA	Higher risk of hospitalization correlates with low income
	Lassale et al.^60^	England	
	Patel et al.^61^	England	

## 8 EDUCATION

Education is a social determinant of health that holds significant influence and adaptability. Previous studies have established a positive correlation between education and overall well‐being. Furthermore, recent research has confirmed that education is a crucial factor in determining one's health, as it is linked to life expectancy, morbidity, and health behavior. Education is widely recognized as a crucial factor in reducing poverty and addressing socioeconomic and political disparities. Moreover, prior studies on chronic illnesses have shown that insufficient rates of morbidity and mortality are widespread in different communities. The recent pandemic outbreak has further highlighted the crucial role of education and schools in societies. Evidence suggests that educational attainment is linked to health through several interrelated pathways.^62^ The correlation between an individual's SES and their life expectancy and mortality has been extensively documented. SES is typically determined by factors such as occupation, income, and educational attainment. Studies have shown that individuals with lower SES are at a higher risk of contracting and experiencing more severe symptoms from the prevalence virus.^63^ Moreover, a recent publication has emphasized the indirect impact of lower education levels on the severity of pandemic rates due to several risk factors, such as unhealthy behaviors, poor dietary habits, and smoking.^36^ However, the recent pandemic has led to troubles in children's education in many countries^64^ with remote teaching replacing in‐person teaching and presenting new challenges, such as ensuring access to digital learning devices.^65^ Recent data from the United States suggests that individuals with higher income and education levels have experienced lower mortality rates from COVID‐19 in comparison to those with median income levels.

### 8.1 Gap in current lectures

During the intermission between lectures, it was emphasized that the COVID‐19 pandemic has exacerbated the already widening gaps in inequality and labor market crises. Although current research emphasizes the link between socio‐environmental inequalities at an individual level, the absence of comprehensive data makes it challenging to assess the overall condition of each region worldwide. The present study provides a description of the region, but the information provided is not entirely clear. Further examination is necessary to fully understand the impact of various forms of social inequality, especially on vulnerable groups such as women, youth, elderly individuals, persons with disabilities, indigenous communities, and refugee workers. While there is some fundamental environmental information available on the lack of access to safe water, clean air, and waste management in various regions globally, no research has yet explored the correlation between COVID‐19 cases and environmental inequity. However, the evidence suggests that areas with inadequate social and environmental conditions had a higher incidence and prevalence of reported COVID‐19 cases compared to other regions. This gap is particularly significant in impoverished regions, such as those in Asia, Africa, and Latin America.

## 9 CONCLUSION

In this review study, we summarize the social and environmental factors that can directly influence the severity of COVID‐19 incidence and mortality cases, leading to inequities. On the other hand, the negative aspects of environmental injustices related to water, air, and waste management were discussed, as well as the impact of social inequalities in education and the economy on the incidence and mortality of the COVID‐19 pandemic. In summary, COVID‐19 has brought to light the disparities in equal opportunities, and socio‐environmental metrics vary significantly around the globe. This has resulted in a disproportionate impact on developing countries, especially in Africa and certain parts of Asia, where access to healthcare and basic necessities of life is not guaranteed. The existence of socio‐environmental disparities, such as exposure to pollution, lack of access to safe water, inadequate management of wastewater, poor quality housing, overcrowding, and low SES, has been associated with higher COVID‐19 mortality rates globally. Therefore, it is imperative to prioritize the adoption of the latest socio‐environmental information to reduce mortality rates in vulnerable communities. This review emphasizes the importance of closely monitoring populations that are exposed to high environmental risk factors during pandemics. Furthermore, it highlights that socio‐environmental risk factors are likely to worsen in the future unless significant policy improvements are implemented and prompt actions are taken.

## AUTHOR CONTRIBUTIONS

**Laleh R. Kalankesh**: Writing—original draft. **Zahed Rezaei**: Writing—review and editing. **Ali Mohammadpour**: Data curation; project administration. **Mahmud Taghavi**: Investigation; project administration. All authors participated in this research paper.

## CONFLICT OF INTEREST STATEMENT

The authors declare no conflict of interest.

## TRANSPARENCY STATEMENT

The lead author Ali Mohammadpour, Mahmoud Taghavi affirms that this manuscript is an honest, accurate, and transparent account of the study being reported; that no important aspects of the study have been omitted; and that any discrepancies from the study as planned (and, if relevant, registered) have been explained.

## Data Availability

All data relevant to the study are included in the article.
